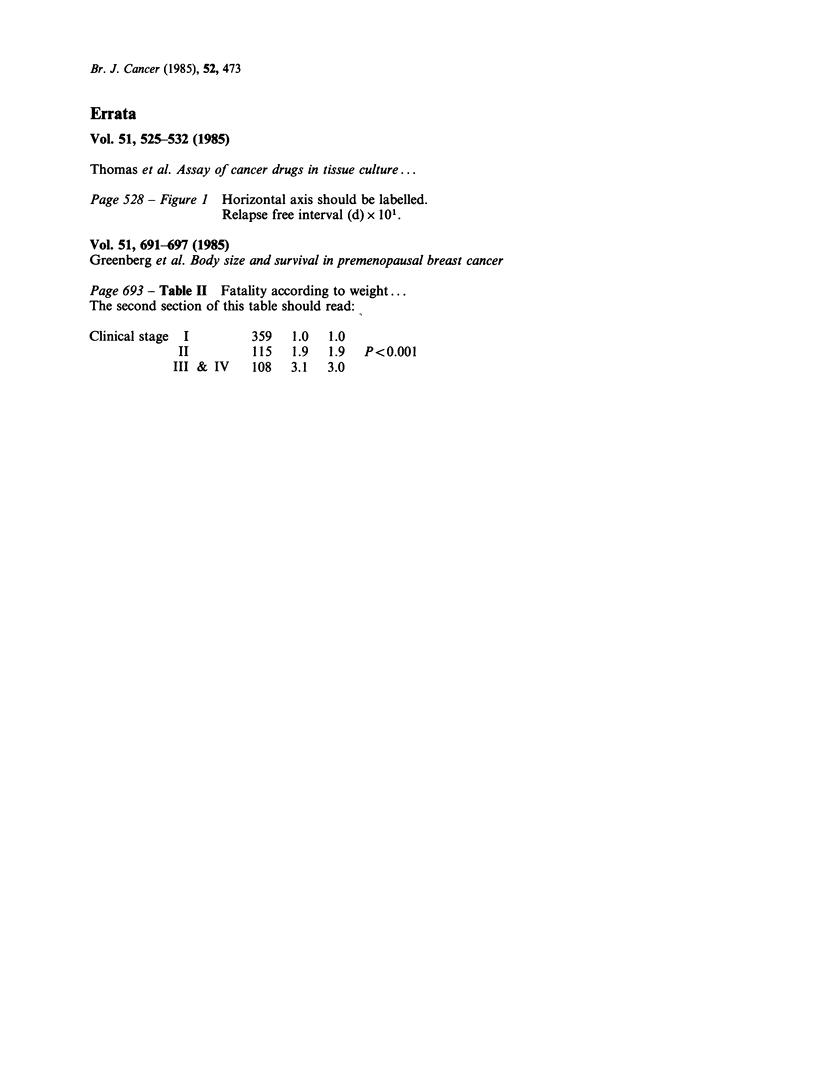# Errata

**Published:** 1985-09

**Authors:** 


*Br. J. Cancer* (1985), **52**, 473

### Errata

**Vol. 51, 525–532 (1985)**

Thomas *et al. Assay of cancer drugs in tissue culture...*

*Page 528 – Figure 1* Horizontal axis should be labelled.
Relapse free interval (d) × $10^1$.

**Vol. 51, 691–697 (1985)**
Greenberg *et al. Body size and survival in premenopausal breast cancer*

*Page 693* – **Table II** Fatality according to weight...
The second section of this table should read:

| | | | | | |
|---|---|---|---|---|---|
| Clinical stage | I | 359 | 1.0 | 1.0 | |
| | II | 115 | 1.9 | 1.9 | $P<0.001$ |
| | III & IV | 108 | 3.1 | 3.0 | |